# Molecular epidemiology of carbapenem-resistant *Klebsiella pneumoniae* ST15-KL19 isolates from ICU patients in a Chinese tertiary hospital

**DOI:** 10.3389/fmicb.2025.1673965

**Published:** 2025-11-18

**Authors:** Qiang Xie, Tiantian Xu, Ling Chen, Chunhong Shi

**Affiliations:** Department of Laboratory Medicine, The Affiliated Chuzhou Hospital of Anhui Medical University (The First People's Hospital of Chuzhou), Chuzhou, Anhui, China

**Keywords:** *Klebsiella pneumoniae*, carbapenem resistance, whole-genome sequencing (WGS), antibiotic resistance genes, multilocus sequence typing (MLST)

## Abstract

Carbapenem-resistant *Klebsiella pneumoniae* (CRKP) represents a growing threat in healthcare settings, with sequence type 15 (ST15) increasingly recognized as a high-risk lineage. To characterize its molecular and epidemiological features in eastern China, we investigated 17 non-duplicate ST15 CRKP isolates obtained from patients in the intensive care unit (ICU) of a tertiary hospital in Chuzhou. Whole-genome sequencing, antimicrobial susceptibility testing, resistance and virulence gene profiling, plasmid analysis, and core genome multilocus sequence typing with SNP analysis were performed. All isolates belonged to the ST15-KL19 clone and carried a conserved set of resistance genes, including aminoglycoside-modifying enzymes (aac(3)-IId, aac(6′)-Ib-cr), extended-spectrum β-lactamases (blaCTX-M-15, blaSHV-106, blaTEM-1B), carbapenemase (blaKPC-2), oxacillinase (blaOXA-1), and additional determinants such as fosA, oqxA, and oqxB. The isolates exhibited complete resistance to β-lactams and carbapenems but remained susceptible to tigecycline and amikacin. Two predominant virulence gene patterns were identified, with most isolates harboring fimbrial operons, siderophore systems, and T6SS components. Plasmid analysis revealed the presence of IncFIB(K) and repB(R1701). Phylogenetic analysis demonstrated three clusters with strong spatiotemporal overlap, supporting nosocomial transmission. Clinically, infections were associated with prolonged ICU stays and poor outcomes, with only 17.6% of patients achieving full recovery and 5.9% experiencing in-hospital mortality. These findings indicate that ST15-KL19 CRKP constitutes a regionally endemic clone with high resistance and virulence potential, underscoring the urgent need for strengthened molecular surveillance and stringent infection control measures.

## 1 Introduction

Carbapenem-resistant *Enterobacterales* (CRE) are defined, according to the Clinical and Laboratory Standards Institute (CLSI) criteria [Clinical and Laboratory Standards Institute (CLSI), 2025], as *Enterobacterales* that are non-susceptible to at least one carbapenem agent (imipenem, meropenem, doripenem, or ertapenem). CRE have emerged as a major global health threat due to their limited therapeutic options, high mortality rates, and frequent association with hospital outbreaks.

Among CRE, CRKP has attracted particular attention as a predominant pathogen worldwide. CRKP exhibits high resistance to most *β*-lactam antibiotics and has the ability to cause severe infections, particularly in immunocompromised patients and those in ICUs (Karampatakis et al., 2023; Li et al., 2024). In China, the CRKP epidemic has been largely dominated by sequence type 11 (ST11), a hyperendemic clone responsible for numerous hospital outbreaks and associated with a wide array of resistance determinants (Zhou et al., 2023; Jiang et al., 2025). However, in recent years, ST15, another high-risk international clone, has been increasingly reported in various clinical settings across China and other countries, often carrying the carbapenemase gene *blaKPC-2* and multiple virulence factors (Song et al., 2023; Martins et al., 2020; Gato et al., 2023).

Unlike ST11, ST15 has historically been less prevalent in China. Nonetheless, its rising detection rate, together with its frequent association with multidrug resistance and hypervirulence, suggests that ST15 may soon become a dominant CRKP lineage in high-risk hospital environments such as ICUs. Notably, capsular serotype KL19 has recently emerged among ST15 strains and is thought to enhance bacterial survival, immune evasion, and transmission capacity (Feng et al., 2023; Zhao et al., 2022).

Despite growing concern, molecular epidemiological data on ST15-KL19 CRKP remain scarce in China, particularly in second- and third-tier cities where genomic surveillance is limited. To address this gap, we comprehensively characterized ST15-KL19 CRKP isolates collected from ICU patients at a tertiary hospital in Chuzhou, Anhui Province. Seventeen non-duplicate isolates underwent antimicrobial susceptibility testing and whole-genome sequencing (WGS). We further analyzed their resistance gene profiles, virulence gene repertoires, plasmid replicon types, and clonal relationships using core genome multilocus sequence typing (cgMLST). In addition, relevant clinical information, including ICU length of stay, infection outcomes, and patient comorbidities—was collected to evaluate the potential clinical impact and pathogenicity of this emerging clone. This study aims to provide insights into both the molecular epidemiology and clinical significance of ST15-KL19 CRKP and to inform targeted strategies for infection control and antimicrobial stewardship in ICU settings.

## 2 Materials and methods

### 2.1 Bacterial isolates and clinical data collection

A total of 17 non-duplicate *CRKP* isolates were obtained from patients admitted to the ICU of a tertiary hospital in Chuzhou, Anhui Province, China, between May and November 2020. Relevant clinical data were retrospectively retrieved from electronic medical records, including age, sex, comorbidities, type of infection, length of ICU stay, prior antimicrobial exposure, and clinical outcome (survival or death). The study was conducted in accordance with hospital ethical guidelines, and patient identifiers were anonymized.

### 2.2 Bacterial identification and antimicrobial susceptibility testing

All isolates were identified and tested for antimicrobial susceptibility using the VITEK 2-Compact automated system (bioMérieux, France) with the corresponding AST cards. Antimicrobial susceptibility results were interpreted according to the latest CLSI breakpoints (M100, 35th edition, 2025) [Clinical and Laboratory Standards Institute (CLSI), 2025]. Quality control strains included *Escherichia coli* ATCC 25922 and *Klebsiella pneumoniae* ATCC 700603. The antibiotics included amoxicillin/clavulanic acid, piperacillin/tazobactam, cefoperazone/sulbactam, cefuroxime, cefuroxime axetil, cefoxitin, ceftriaxone, ceftazidime, cefepime, ertapenem, imipenem, levofloxacin, amikacin, trimethoprim/sulfamethoxazole, and tigecycline. Culture media were purchased from Antu Bioengineering Co., Ltd. (Zhengzhou, China).

### 2.3 Whole genome sequencing

Genomic DNA was extracted using a commercial bacterial genomic DNA extraction kit (Tiangen Biotech, Beijing, China). Sequencing was performed by NovoGene Bioinformatics Technology Co., Ltd. (Beijing, China). A 350 bp paired-end library was constructed for each isolate and sequenced using the Illumina platform to obtain a minimum of 100 × clean data coverage. *De novo* genome assembly was carried out using SPAdes version 3.15 (Bankevich et al., 2012), and assemblies were quality-assessed with QUAST (Gurevich et al., 2013). Genome size and GC content were calculated.

### 2.4 Annotation of antimicrobial resistance and virulence genes

Bioinformatic analyses were conducted on the assembled genome sequences using the BacWGSTdb platform (http://bacdb.cn/BacWGSTdb/) for preliminary prediction of antimicrobial resistance and virulence genes. Antimicrobial resistance genes were further identified using two public databases: the Antibiotic Resistance Genes Database (ARDB) and the Comprehensive Antibiotic Resistance Database (CARD). The DIAMOND software was used to align isolate-derived amino acid sequences against the ARDB database for functional resistance annotation. The Resistance Gene Identifier (RGI) tool from CARD was applied with its built-in BLASTp algorithm and a default E-value threshold of ≤1e-30. Database access: ARDB (http://ardb.cbcb.umd.edu/), CARD (https://card.mcmaster.ca/), DIAMOND (http://www.diamondsearch.org) (Liu and Pop, 2009; Jia et al., 2024; Buchfink et al., 2021). Virulence-associated genes were identified using the Virulence Factors Database (VFDB, http://www.mgc.ac.cn/VFs/) through the Abricate pipeline (https://github.com/tseemann/abricate). Matches with ≥90% sequence identity and ≥80% coverage were considered positive. Virulence gene clusters of particular relevance, including ybt (yersiniabactin), ent/fep (enterobactin), mrk/fim (type I and type III fimbriae), capsule synthesis loci (wzi/wzm/wzt), and T6SS-related genes (hcp/tssD, vipB/tssC), were specifically screened and annotated for each isolate.

### 2.5 Multilocus sequence typing (MLST)

MLST was performed based on the sequences of seven housekeeping genes, and sequence types (STs) were determined using the *Klebsiella pneumoniae* MLST database hosted on PubMLST^1^ (Jolley et al., 2018).

### 2.6 Clonal relatedness and plasmid analysis

Clonal relatedness of the 17 ST15 CRKP isolates was assessed using the Pathogenwatch online platform^2^ (Argimón et al., 2020) which also identified their plasmid replicon types. To assess the genomic relatedness of our isolates in a broader context, we retrieved representative ST15 CRKP genomes from public databases, including isolates from Anhui Province (the study site) and its neighboring provinces (e.g., Zhejiang, Shanghai) and from selected countries in Europe and South America. A core-genome MLST (cgMLST) analysis was performed, and a minimum spanning tree (MST) was generated to visualize the clonal relationships (Supplementary Figure 1).

### 2.7 Data analysis

Antimicrobial susceptibility data and isolate distribution were analyzed using WHONET version 5.6 software.

## 3 Results

### 3.1 Clinical characteristics of ST15 CRKP-infected ICU patients

WGS analysis revealed that all 17 CRKP isolates recovered from ICU patients belonged to sequence type 15 (ST15). The clinical characteristics of the 17 ICU patients infected with ST15 CRKP are summarized in Table 1 and further detailed on a case-by-case basis in Supplementary Table 1. The mean age was 60.6 years, with a wide age range spanning from young adults to elderly individuals. The majority of patients were male. Notably, all patients had received antibiotics within the three months prior to CRKP isolation and had undergone invasive respiratory procedures. A substantial proportion had multiple comorbidities, most commonly neurological disorders and pulmonary infections. Other frequent conditions included hypertension, trauma-related injuries, coronary artery disease, and diabetes mellitus.

**Table 1:** Clinical characteristics of the 17 ICU patients infected with ST15 CRKP.

Variable	Value
Total number of patients	17
Age (years)	60.6 ± 15.6 (range: 28–82)
Sex	
Male	14 (82.4%)
Female	3 (17.6%)
History of antibiotic use (within 3 months)	17 (100%)
Invasive respiratory procedures	17 (100%)
Comorbidities	
Neurological diseases^a^	16 (94.1%)
Pulmonary infections	15 (88.2%)
Hypertension	8 (47.1%)
Diabetes mellitus	4 (23.5%)
Coronary artery disease	5 (29.4%)
Trauma-related injuries^b^	5 (29.4%)
Renal failure or Urinary tract infection	3 (17.6%)
Septic shock	2 (11.8%)
Common antibiotics used^c^	
Piperacillin/tazobactam	11 (64.7%)
Linezolid	13 (76.5%)
Ceftriaxone	10 (58.8%)
Meropenem	5 (29.4%)
Imipenem/cilastatin	4 (23.5%)
Tigecycline	4 (23.5%)
Levofloxacin	3 (17.6%)
Amoxicillin-clavulanate	2 (11.8%)
Voriconazole	2 (11.8%)

a Neurological diseases include cerebral infarction, brain hemorrhage, brain herniation, subarachnoid hemorrhage, and traumatic brain injury, etc.

b Trauma includes fractures, contusions, hemothorax, open wounds, and other associated injuries.

c Only antibiotics used in ≥2 patients are listed.

Linezolid, piperacillin/tazobactam, and ceftriaxone were the most frequently administered antibiotics prior to isolation. These patterns reflect a patient population with severe underlying disease and extensive antimicrobial exposure.

### 3.2 ICU stay, specimen types, and clinical outcomes

The majority of isolates were recovered from sputum specimens (94.1%, 16/17), with only one from blood (5.9%). The ICU stay duration among the patients ranged from 10 to 51 days, with a median of approximately 26 days. Regarding clinical outcomes, 8 patients (47.1%) showed clinical improvement, 3 (17.6%) achieved full recovery, and 1 (5.9%) died during hospitalization. The remaining 5 patients (29.4%) were classified as “not resolved” or “other.” These findings reflect the severe nature of ST15 CRKP infections, which often necessitate prolonged ICU care and are associated with unfavorable outcomes in a considerable proportion of patients (see Table 2).

**Table 2:** ICU stay, specimen type, and clinical outcomes.

Strain ID	Age	Gender	ICU Stay (days)	Specimen type	Clinical outcome
K2.01	76	M	35	Sputum	Death
K2.02	56	M	30	Sputum	Improved
K2.03	67	M	23	Sputum	Improved
K2.05	41	M	23	Sputum	Cured
K2.06	28	F	46	Sputum	Other
K2.13	63	M	51	Blood	Improved
K2.14	50	M	19	Sputum	Cured
K2.15	66	M	50	Sputum	Cured
K2.16	52	M	41	Sputum	Improved
K2.17	51	M	26	Sputum	Improved
K2.19	80	F	24	Sputum	Other
K2.20	82	M	50	Sputum	Other
K2.21	76	M	33	Sputum	Other
K2.23	66	M	10	Sputum	Improved
K2.24	72	F	14	Sputum	Not resolved
K2.26	67	M	10	Sputum	Improved
K2.28	37	M	18	Sputum	Improved

### 3.3 Antibiotic susceptibility profiles of ST15 CRKP isolates

**Figure 1:**
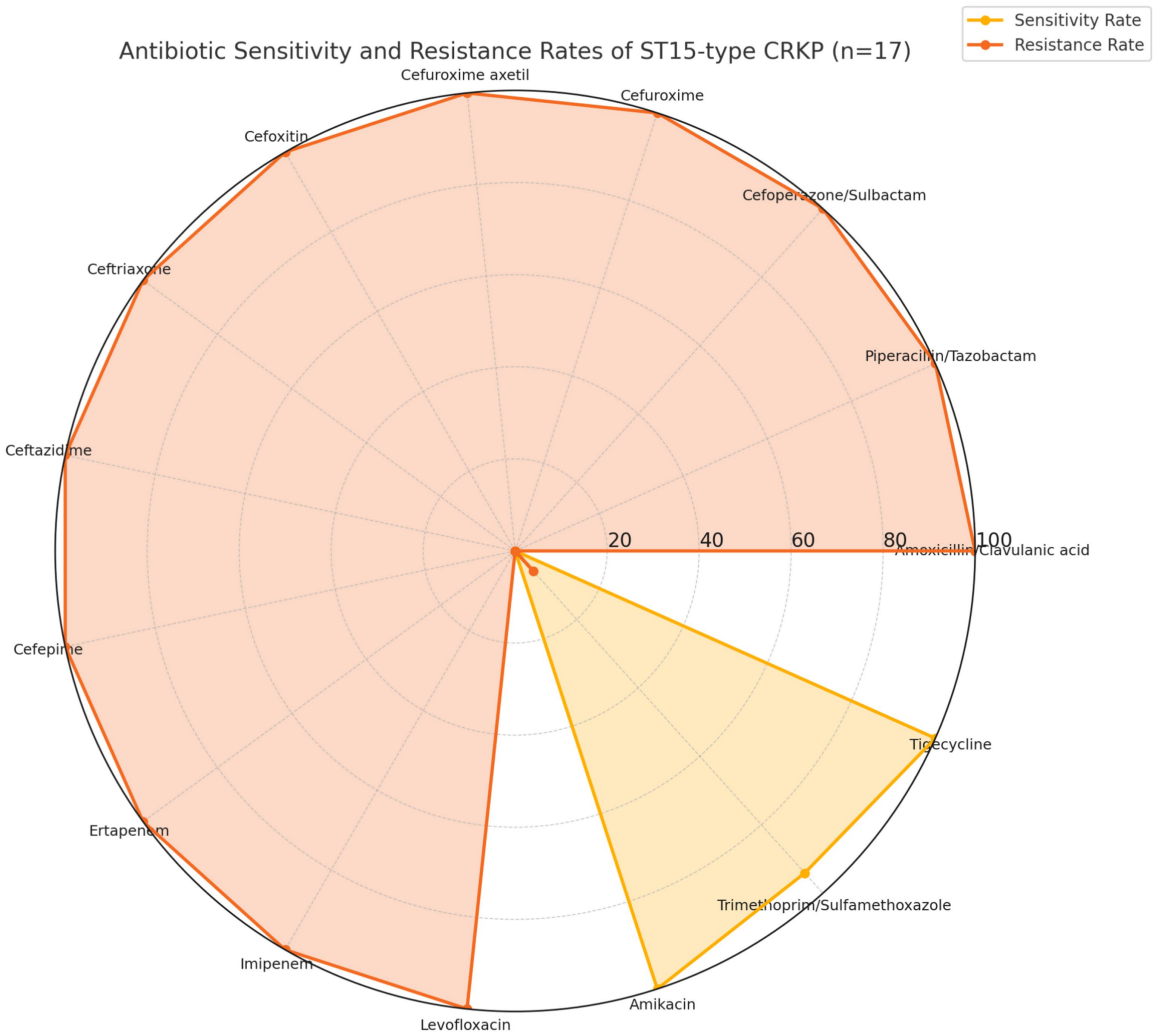
Radar chart showing the resistance rates of 17 ST15 CRKP isolates to 15 tested antibiotics. Each axis represents a tested antibiotic, and the plotted points correspond to resistance to *β*-lactam antibiotics, while tigecycline and amikacin retained full activity (0% resistance).

The susceptibility and resistance patterns of the 17 isolates are summarized in Figure 1. Using the latest CLSI breakpoints (M100-Ed35, 2025), nearly all isolates were resistant to *β*-lactam and carbapenem antibiotics, while all remained fully susceptible to tigecycline and amikacin (100%). Trimethoprim/sulfamethoxazole showed partial activity with a resistance rate of 5.9%, indicating limited therapeutic options. The full susceptibility data, including the cefepime SDD category defined by CLSI 2025 (no isolates fell into SDD category in this cohort), are provided in Supplementary Table 2. Tigecycline susceptibility was interpreted according to FDA criteria.

Among the ICU patients, most initially received empirical carbapenem- or *β*-lactam/β-lactamase inhibitor–based regimens, which were largely ineffective due to the extensive resistance profiles of ST15-KL19 isolates. In contrast, improved clinical outcomes were more often observed in patients who received tigecycline- or amikacin-containing regimens, consistent with the *in vitro* susceptibility results (Supplementary Table 2).

### 3.4 Resistance genes, virulence genes, and capsular serotype

All 17 ST15 CRKP isolates shared the same capsular serotype KL19. Comprehensive genomic analysis revealed that all isolates carried a consistent set of resistance genes, including aminoglycoside-modifying enzymes (*aac(3)-IId*, *aac(6′)-Ib-cr*), *β*-lactamases (*blaCTX-M-15*, *blaSHV-106*, *blaTEM-1B*), carbapenemase (*blaKPC-2*), and oxacillinase (*blaOXA-1*). Additionally, quinolone resistance determinants (*oqxA*, *oqxB*) and the fosfomycin resistance gene (*fosA*) were universally detected.

Virulence gene analysis revealed that all 17 ST15-KL19 CRKP isolates harbored a broad array of virulence factors associated with adhesion, iron acquisition, biofilm formation, capsule synthesis, and type VI secretion system (T6SS). All strains carried complete *mrkA*, *mrkB*, *mrkC*, *mrkD*, *mrkF*, *mrkH*, *mrkI*, *mrkJ* (type III fimbriae) and *fimA*, *fimB*, *fimC*, *fimD*, *fimE*, *fimF*, *fimG*, *fimH*, *fimI*, *fimK* (type I fimbriae) operons, suggesting strong adhesive and biofilm-forming capacities. Iron acquisition genes were universally present, including *entA*, *entB*, *entC*, *entE*, *entF* (enterobactin biosynthesis), *fepA*, *fepB*, *fepC*, *fepD*, *fepG* (ferric enterobactin transporters), and *ybtA*, *ybtE*, *ybtP*, *ybtQ*, *ybtS*, *ybtT*, *ybtU*, *ybtX*, *irp1*, *irp2*, *fyuA* (yersiniabactin system). Based on virulence gene composition, isolates were divided into two major patterns: Pattern I (*n* = 15): carried a complete virulence repertoire including fimbrial clusters (*fim*, *mrk*), siderophore systems (*ent*, *fep*, *ybt*), T6SS components (*hcp*, *vipA–B*, *tssK*), and capsule synthesis genes (*wzi*, *wzm*, *wzt*); Pattern II (*n* = 2): similar to Pattern I but lacked *ybtX*, indicating a partial deletion in the yersiniabactin operon.

**Figure 2:**
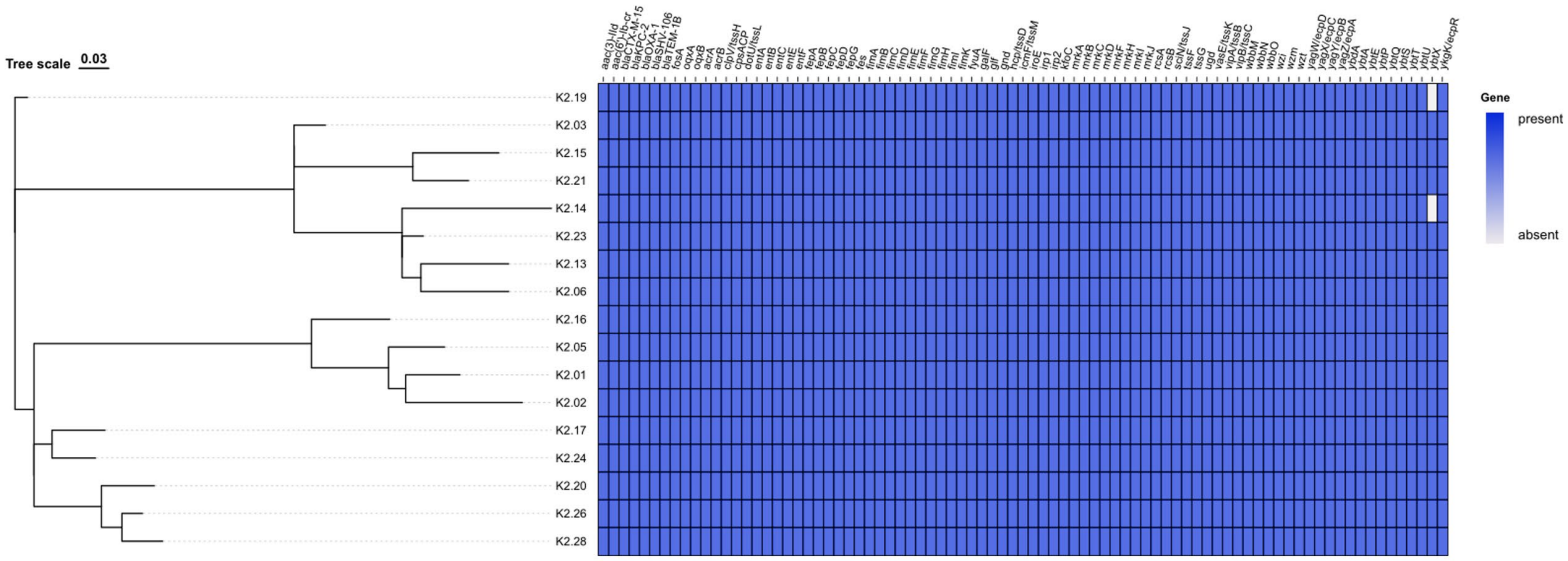
Distribution of resistance genes, virulence genes, and plasmid types in 17 ST15 CRKP isolates. Genes are shown along the x-axis and isolates are clustered on the y-axis based on whole-genome similarity. Each filled blue block represents the presence of a gene; lighter blocks indicate absence. Resistance genes include *blaKPC-2*, *blaCTX-M-15*, *blaSHV-106*, etc.; virulence genes include *mrk* operon, *ybt* cluster, and *ent* cluster. Hypervirulence markers were not detected.

In addition, all isolates harbored multidrug efflux pump genes *acrA* and *acrB*, which may enhance resistance via active drug efflux. These findings indicate that the isolates carried different subsets of virulence genes rather than all possible variants, highlighting intra-clonal diversity within ST15-KL19 CRKP. However, none of the isolates harbored hypervirulence-associated genes such as *iucA*, *iroB*, *peg-344*, *rmpA*, and *rmpA2*. Plasmid replicon analysis showed that all strains carried IncFIB(K) and repB(R1701) plasmid types. A summary of resistance and virulence gene profiles is presented in Figure 2.

### 3.5 Phylogenetic relatedness and clonal transmission of ST15 CRKP isolates

**Figure 3:**
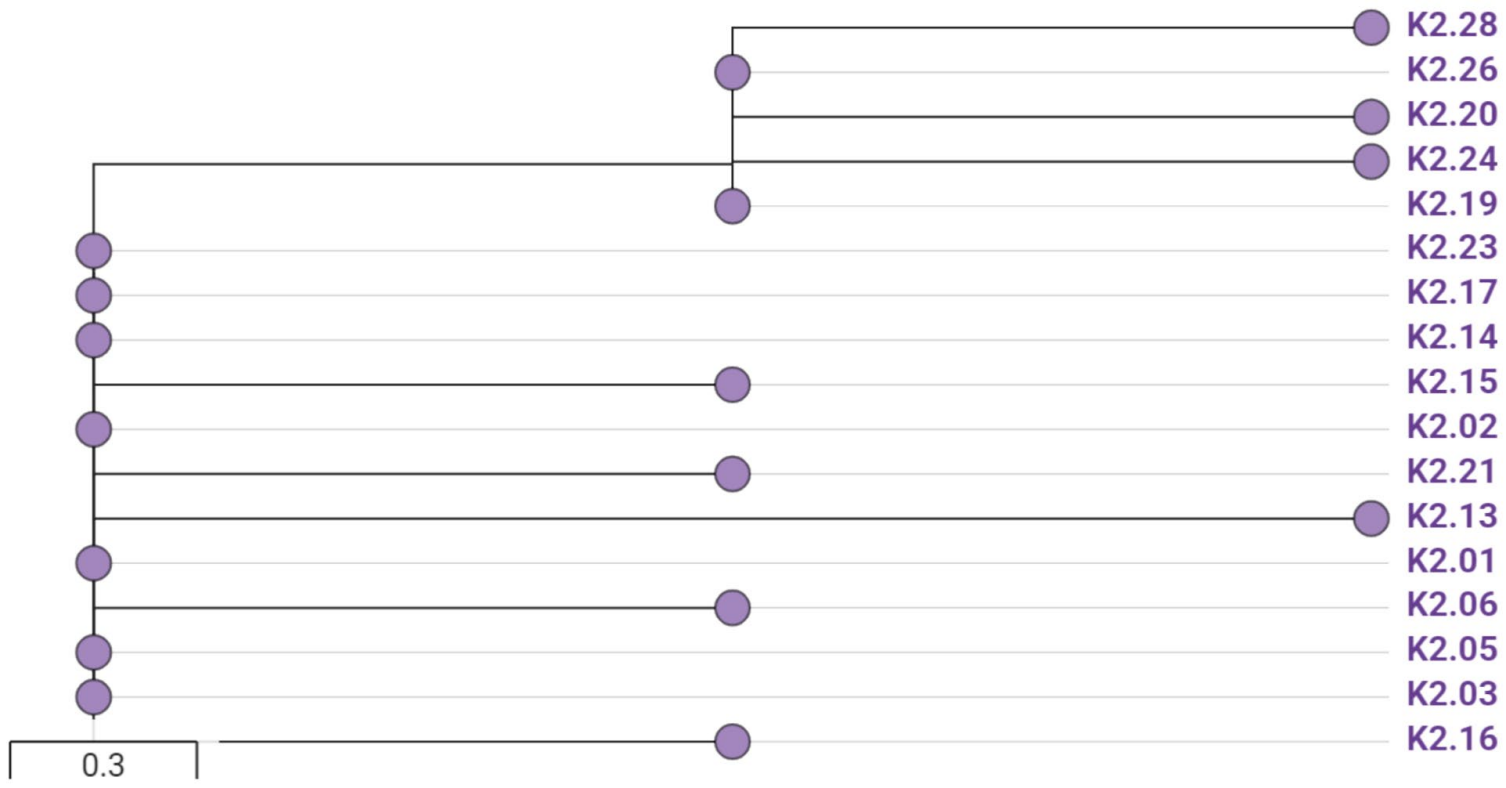
Minimum spanning tree based on cgMLST profiles of 17 ST15 CRKP isolates. Isolates were grouped into three clusters (from left to right: A, B, and C). Nodes represent isolates, and edge lengths reflect allelic differences. Temporal and spatial overlaps suggest possible nosocomial transmission.

The phylogenetic analysis of the 17 ST15 CRKP isolates using cgMLST revealed three distinct clusters (from left to right): Cluster A (7 isolates), Cluster B (6 isolates), and Cluster C (4 isolates) (Figure 3). Among the strains, 16 shared a cgMLST type of 2 cc9, while one isolate (K2.xx) was typed as ed2d. All isolates possessed the O1/O2v2 O-antigen locus. The cgMLST-based MST further demonstrated that these isolates formed a tight clonal cluster and showed the closest genomic relatedness to a representative strain from Anhui Province, rather than to strains from other provinces or countries, suggesting possible regional dissemination (Supplementary Figure 1).

Spatiotemporal mapping of bed locations and isolation periods revealed notable overlaps within each cluster, suggesting potential nosocomial transmission events (Supplementary Table 3). In Cluster A, five of the seven isolates showed overlapping ICU stays, and three were isolated from adjacent beds; notably, K2.02 and K2.05 originated from the same bed (Bed 5). In Cluster B, four isolates shared overlapping timeframes, and K2.06 and K2.19 were obtained from the same bed at different times. Cluster C showed similar patterns, with three isolates (K2.20, K2.24, and K2.28) overlapping in time, while K2.13 and K2.24 were isolated from the same bed despite temporal separation. These findings provide strong support for clonal dissemination within the ICU setting.

**Figure 4:**
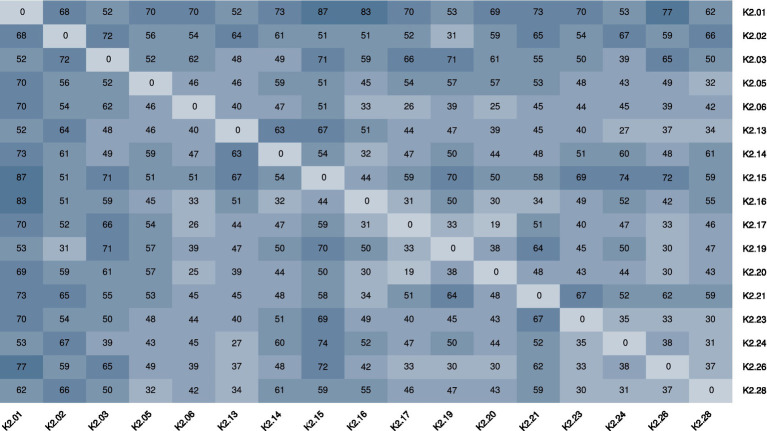
SNP distance analysis of 17 ST15 CRKP isolates. Pairwise SNP distances <50 were considered indicative of high genomic similarity. Closely related isolates are highlighted, suggesting clonal dissemination within the ICU setting.

SNP distance analysis further confirmed the close genetic relatedness among multiple isolates. A pairwise SNP distance of <50 was considered indicative of high homology. Strains such as K2.28 and K2.03, K2.05, K2.06, K2.13, K2.17, K2.19, K2.20, K2.23, K2.24, and K2.26 met this criterion. In addition, K2.02 and K2.19, as well as K2.14 and K2.16, are also highly homologous, respectively. These strongly support recent transmission events. Detailed SNP clustering is shown in Figure 4.

## 4 Discussion

In recent years, the detection rate of CRE has been increasing steadily in clinical settings, particularly in ICUs, where CRKP has emerged as a major threat. In our hospital, the CRKP detection rate in the ICU rose from 10.7% in 2018 to 36.4% in 2021, significantly higher than the national surveillance data from CARSS during the same period (6.4% in 2014 to 10% in 2019, with an ICU-specific rate of 23.0% in 2019) (Wu et al., 2021). This sharp increase suggests a substantial risk of nosocomial transmission. WGS identified 17 ST15-KL19 CRKP isolates with overlapping hospital stays, shared bed locations, and high genomic homology, indicating a localized clonal outbreak within the ICU.

ST15 is emerging as a high-risk clone of CRKP in China, with increasing reports suggesting regional expansion and partial replacement of the dominant ST11 clone in some settings (Chen et al., 2021; Liu et al., 2024). Internationally, ST15 has also been identified in countries such as Italy, Brazil, UK, Portugal (Martins et al., 2020; Sacco et al., 2022; Davies et al., 2017; Spadar et al., 2022). In our study, all 17 ST15 isolates were recovered from ICU patients, primarily from sputum samples (16/17), suggesting respiratory tract infections as the main source. Most patients were male, had at least three underlying conditions, experienced prolonged hospital stays, had received antibiotic treatment, and underwent invasive procedures—all recognized risk factors for CRKP colonization and transmission. The open layout of our ICU, lacking physical partitions and involving frequent patient contact, long hospital stays, and intensive interventions, likely facilitated bacterial spread. Genomic clustering revealed close associations between isolates and bed locations/ hospitalization periods, supporting potential transmission events. For instance, K2.06 and K2.19 were recovered from Bed 3, and K2.13 and K2.24 from Bed 2, suggesting possible environmental contamination or asymptomatic colonized hosts. cgMLST and SNP analyses further confirmed the existence of a clonal spread within the ICU.

The cgMLST-based MST demonstrated that our ICU isolates formed a tight clonal cluster and showed the closest genomic relatedness to a representative strain from Anhui Province, rather than to strains from other provinces or countries, suggesting possible regional dissemination (Supplementary Figure 1). At the same time, all isolates in this study shared uniform molecular features, including the KL19 capsular type and carriage of IncFIB(K) and repB(R1701) plasmids, which together constitute a regional molecular signature distinct from other reports. These observations indicate that, although our isolates belong to a clonally disseminated lineage within Anhui, distinct evolutionary trajectories of ST15 CRKP may still exist within different areas of the same province. Moreover, unlike ST15 isolates reported in Iran (Saki et al., 2022), where blaNDM or blaOXA-48-like carbapenemases are frequently detected, our strains consistently carried blaKPC-2, suggesting the emergence of a regionally confined ST15 subclone in eastern China with a distinct resistance profile. cgMLST and SNP analyses grouped the isolates into three clusters, with several pairs differing by fewer than 50 SNPs. Coupled with overlapping hospitalization and bed assignments, this supports nosocomial transmission (see Supplementary Table 3).

CRKP exhibits complex resistance mechanisms, primarily due to carbapenemase production, but also involving AmpC enzymes, extended-spectrum *β*-lactamases (ESBLs), efflux pumps, and porin loss (Hu et al., 2020; Cienfuegos-Gallet et al., 2022; Lee et al., 2023). All 17 isolates carried five β-lactamase genes (*blaCTX-M-15*, *blaKPC-2*, *blaOXA-1*, *blaSHV-106*, and *blaTEM-1B*), consistent with previous reports (Zhao et al., 2022). Fosfomycin is recommended as a therapeutic option for CRE infections in Chinese expert consensus guidelines (Chinese expert consensus on the diagnosis, treatment, and prevention of infections caused by carbapenem-resistant Enterobacteriaceae, 2021). While domestic studies report a fosfomycin resistance rate of 18.7% in CRKP (Wang et al., 2021), foreign data suggest over 90% susceptibility (Zarakolu et al., 2022). However, our isolates all harbored *fosA6*, indicating intrinsic resistance. Although drug susceptibility testing was not performed, clinicians should use fosfomycin cautiously. No resistance genes for tigecycline or polymyxins were detected, indicating these agents may remain viable treatment options. In addition to plasmid-mediated resistance determinants, chromosomal mutations—such as alterations in *ompK36* and *ompK35* leading to reduced membrane permeability, or mutations in *pmrB* contributing to colistin resistance—are also known to play important roles in CRKP resistance. Since chromosomal mutation analysis was not performed in this study, these mechanisms may represent undetected contributors to resistance in our isolates. This limitation warrants further investigation in future studies.

In terms of therapeutic implications, our isolates retained susceptibility to tigecycline and amikacin, which remain important options in clinical practice. Combination therapies, such as tigecycline- or aminoglycoside-based regimens, have been suggested to improve outcomes in patients with severe CRKP infections. Ceftazidime-avibactam has demonstrated high efficacy against CRKP producing *KPC* and *OXA-48* carbapenemases, with superior outcomes compared to traditional regimens (e.g., polymyxins) and significantly reduced nephrotoxicity, and has become one of the preferred treatment options for *KPC*-producing CRKP infections. Meropenem-vaborbactam has also shown excellent activity and safety in the treatment of *KPC*-producing CRKP, including complicated urinary tract infections and bloodstream infections. Cefiderocol, a novel siderophore cephalosporin functioning as a ‘Trojan horse’ by exploiting bacterial iron uptake systems, is active against nearly all types of carbapenemases, including *KPC*, *OXA-48*, and *metallo-β-lactamases*. In our hospital, ceftazidime-avibactam has already been introduced and is expected to play a crucial role in the management of *KPC*-producing CRKP infections. However, meropenem-vaborbactam and cefiderocol were not available in our hospital during the study period, highlighting the need for broader access to these novel agents in China.

Virulence analysis showed that all 17 isolates carried a broad spectrum of virulence genes related to adhesion (*fim/mrk*), iron acquisition (*ent/fep/ybt*), biofilm formation, capsule synthesis (*wzi/wzm/wzt*), and the T6SS system, highlighting strong adaptation to both host environments and hospital settings. All isolates possessed complete type I (*fim*) and type III (*mrk*) fimbrial operons. Type I fimbriae penetrate the capsule to adhere to host cells, while type III fimbriae promote biofilm formation—key factors for colonization and persistent infection. Their coexistence may enhance adhesion and biofilm-forming capacity in the ICU environment, increasing both pathogenicity and treatment difficulty (Beckman et al., 2024; Zhang et al., 2025). The widespread presence of *ent, fep,* and *ybt* genes supports strong iron acquisition ability, enhancing survival in iron-limited conditions like host blood. T6SS genes (e.g., *hcp/tssD*, *vipB/tssC*) suggest potential interspecies competition and immune evasion capabilities.

Several recent studies have reported a similar pattern of overlap between virulence genes (including *ent*, *mrk*, *ybt*) and antibiotic resistance in *K. pneumoniae*. For example, Shrestha et al. found that many MDR isolates from a neonatal intensive care unit in Nepal carried *ent*, *mrk*, and other core/accessory virulence genes (Shrestha et al., 2024). Regulation studies have shown that the *mrkHIJ* operon plays a key role in regulating type-3 fimbriae expression and biofilm formation (Li et al., 2023). Moreover, in Egypt, high-risk carbapenem-resistant clones were found to harbor hypervirulence determinants among clinical *K. pneumoniae* isolates (Abdelsalam et al., 2024). These findings support the genomic detection of *ybt*, *mrk*, and *ent* clusters in the present isolates as potentially meaningful. An important limitation, however, is that virulence was assessed only at the genomic level. Due to biosafety and ethical constraints, no *in vivo* experiments could be performed. Future studies incorporating animal models will therefore be essential to validate the in vivo relevance of these genomic findings.

Notably, 15 isolates possessed a complete virulence profile, while 2 lacked *ybtX*, possibly due to instability of the ICEKp integrative element or selective loss in iron-rich environments. Although these strains demonstrated strong adhesion and colonization potential, no classic hypervirulent markers (e.g., *iucA*, *rmpA*, *peg-344*) were detected, indicating these were MDR + moderate-virulence CRKP strains rather than typical hypervirulent *K. pneumoniae* (hvKP). Similar “non-hvKP with enhanced virulence” characteristics have been reported in other Chinese ST15-*KPC-2* clones (Zhao et al., 2022).

Clinically, the median ICU stay was 26 days; one patient died, and one failed to improve, while others recovered or improved. Poor outcomes were associated with longer ICU stays, suggesting ST15-KL19 CRKP may prolong hospitalization and complicate treatment. Although statistical significance cannot be established due to the small sample size, the trend warrants further attention. In addition, review of treatment records indicated that most patients initially received empirical carbapenem- or *β*-lactam/β-lactamase inhibitor–based regimens, which were largely ineffective due to the extensive resistance profiles. In contrast, improved outcomes were more often observed among patients treated with tigecycline- or amikacin-containing regimens, consistent with the *in vitro* susceptibility results. While the limited number of cases precludes definitive conclusions, these preliminary correlations highlight the potential clinical relevance of treatment choice in ICU settings and underscore the urgent need for effective therapeutic strategies against ST15-KL19 CRKP.

Beyond this, this study has some limitations, including a small sample size, lack of functional validation, and absence of retrospective epidemiological investigation. In addition, chromosomal mutations (e.g., *ompK36*, *ompK35*, *pmrB*) were not analyzed, which may represent undetected resistance mechanisms. Furthermore, while ceftazidime–avibactam was available in our hospital and has been introduced into clinical practice, other novel agents such as meropenem–vaborbactam and cefiderocol were not available during the study period, which may have influenced treatment options. Moreover, the phylogenetic comparison was based on a selection of representative ST15 genomes from public databases, providing a regional and international context but not a comprehensive global picture. As shown in Figure 2, we compared the presence and absence of major resistance and virulence genes among the isolates; however, we did not analyze the structural arrangement of these gene clusters, which has been reported to influence the evolution of high-risk clones (Zhang, D. F., et al., 2022). This limitation should be addressed in future comparative genomic studies. Nonetheless, our findings suggest that ST15-KL19 CRKP poses a quadruple threat—marked by high resistance, enhanced virulence, effective transmissibility, and clinical risk. Enhanced molecular surveillance, resistance mechanism studies, and hospital infection control measures are urgently needed to prevent further dissemination and evolution.

## 5 Conclusion

This study identified clonal dissemination of ST15-KL19 CRKP in the ICU of a hospital in Chuzhou, China. The isolates exhibited high resistance and virulence, all carrying *blaKPC-2* and *fosA6*, with a uniform KL19 capsular type, suggesting a regionally endemic clone. cgMLST and SNP analyses support possible nosocomial transmission. These findings highlight the need for enhanced molecular surveillance and infection control to prevent further spread.

## Data Availability

The original contributions presented in the study are publicly available. The data can be accessed at: https://www.ncbi.nlm.nih.gov/bioproject/PRJNA1315244. The whole‐genome sequencing (WGS) data generated in this study have been deposited in the NCBI Sequence Read Archive (SRA) under BioProject accession number PRJNA1315244. Representative ST15 Klebsiella pneumoniae genomes used for phylogenetic comparison were retrieved from NCBI (BioProject IDs: PRJNA975316, PRJNA823907, PRJNA523565, PRJNA746265, PRJNA540253, PRJEB8009, PRJNA408270).

## Footnotes

^1^ https://pubmlst.org/organisms/klebsiella-pneumoniae

^2^ https://pathogen.watch/
