# Supplementary figures and images for: Molecular epidemiology of carbapenem-resistant Klebsiella pneumoniae ST15-KL19 isolates from ICU patients in a Chinese tertiary hospital
Source: Front Microbiol. 2025 Nov 18;16:1673965. doi: 10.3389/fmicb.2025.1673965 (PMC12671566; doi:10.3389/fmicb.2025.1673965)

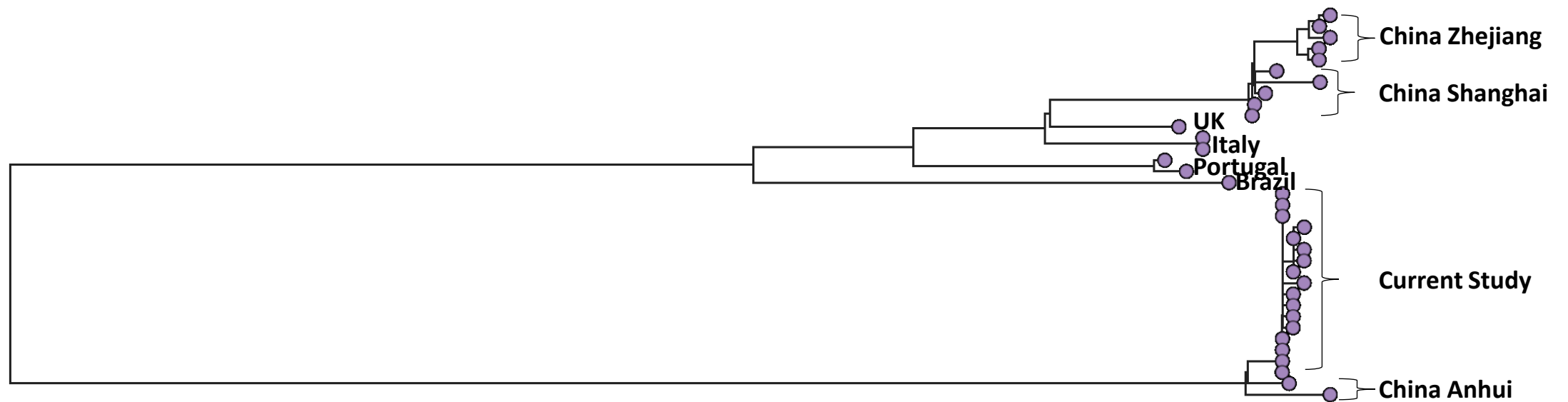

Supplement: Supplementary file 1 [file Data_Sheet_1.PDF]
